# Orthogonal test design for the optimization of superparamagnetic chitosan plasmid gelatin microspheres that promote vascularization of artificial bone

**DOI:** 10.1002/jbm.b.34491

**Published:** 2019-10-12

**Authors:** Chen Tao, Xie Lina, Wang Changxuan, Luo Cong, Yang Xiaolan, Huang Tao, An Hong

**Affiliations:** ^1^ Department of Orthopaedics, Children's Hospital of Chongqing Medical University, Ministry of Education Key Laboratory of Child Development and Disorders, National Clinical Research Center for Child Health and Disorders (Chongqing) China International Science and Technology Cooperation base of Child development and Critical Disorders, Children's Hospital of Chongqing Medical University, Chongqing, P.R China, Chongqing Key Laboratory of Pediatrics Chongqing Engineering Research Center of Stem Cell Therapy; ^2^ Department of Pharmacology Chongqing Medical University, Yuzhong District, Yixueyuan Road1# Chongqing 400016 China; ^3^ Department of Orthopaedics The First Affiliated Hospital of Chongqing Medical University, Yuzhong District, Youyi Road 1# Chongqing 400016 China

**Keywords:** angiogenesis, magnetic field, magnetic gene‐loaded microspheres, VEGF

## Abstract

The optimal conditions for the preparation of superparamagnetic chitosan plasmid (pReceiver‐M29‐VEGF165/DH5a) gelatin microspheres (SPCPGMs) were determined. Then, the performance of the SPCPGMs during neovascularization was evaluated in vivo. The SPCPGMs were prepared through a cross‐linking curing method and then filled into the hollow scaffold of an artificial bone. Neovascularization at the bone defect position was histologically examined in samples collected 2, 4, 6, and 8 weeks after the operation. The cellular magnetofection rate of superparamagnetic chitosan nanoparticles/plasmid (pReceiver‐M29‐VEGF165/DH5a) complexes reached 1–3% under static magnetic field (SMF). Meanwhile, the optimal conditions for SPCPGM fabrication were 20% Fe_3_O_4_ (w/v), 4 mg of plasmid, 5.3 mg of glutaraldehyde, and 500 rpm of emulsification rotate speed. Under oscillating magnetic fields (OMFs), 4–6 μg of plasmids was released daily for 21 days. Under the combined application of SMF and OMF, evident neovascularization occurred at the bone defect position 6 weeks after the operation. This result is expected to provide a new type of angiogenesis strategy for the research of bone tissue engineering.

## 1 INTRODUCTION

Large segmental bone defect is one of the major problems that require urgent clinical solution (Verrier, Alini, Alsberg, et al., 2016; Yassine, Mokhtar, Houari, Karim, & Mohamed, 2017). Tissue engineering is an effective method for bone restoration, and angiogenesis in tissue‐engineered bone is a key factor in the use of this technology in clinical applications (Almubarak et al., 2016; Fan, Crawford, & Xiao, 2010; Jin et al., 2016; Zhao et al., 2017). In vivo, cells that are more than 200 μm away from blood capillaries in artificial bones rarely survive because of insufficient nutrient and oxygen supplies (Lovett, Lee, Edwards, & Kaplan, 2009). Hence, the in vitro structure of a tissue‐engineered bone is insufficiently capable of repairing large bone defects in most cases. Therefore, multiple artificial bone vascularization methods have been adopted, particularly the application of prevascularized engineered bone tissues and a graft combined with cocultured endothelial cells, osteoblasts, and stem cells (Almubarak et al., 2016; Fan, Zeng, Wang, Zhu, & Pei, 2014; Myeroff & Archdeacon, 2011; Zhang et al., 2016). However, this method has a limited clinical application because it requires complex operations and the availability of tissue materials with blood vessels is limited (Remulla et al., 1995). Vascular endothelial growth factor (VEGF) 165 is one of the most abundant and potent angiogenic agents among all VEGF isoforms (Otrock, Makarem, & Shamseddine, 2007; Street et al., 2002; Yang et al., 2014). However, it is deactivated in the presence of enzymes (Raftery, Mencía, Chen, et al., 2017). Given the progress on the research of control release techniques, the slow release of active growth factors is attracting increased attention (Dionigi et al., 2014; Xie et al., 2016). For example, VEGF encapsulated with calcium alginate beads through the extrusion/external gelation method has a constant release rate of 6 ng/ml/day and may be sustained for 14 days (Gu, Amsden, & Neufeld, 2004). A system wherein the controlled release of insulin is regulated by applying oscillating magnetic field (OMF) increased the release of insulin at levels threefold of that of the control group (Finotelli, Da, Sola‐Penna, et al., 2010). Surface cationic magnetic chitosan‐iron oxide nanoparticles can potentially enhance magnetofection efficiency under static magnetic field (SMF; Sohrabijam, Saeidifar, & Zamanian, 2017). A porous nano‐hydroxyapatite/polyamide66 (n‐HA/PA66) composite has been developed in recent years. Yan proved that the porous n‐HA/PA66 composite is biologically safe and exhibits good biocompatibility, osteoinduction, and osseointegration (Xiong et al., 2014). Meanwhile, the use of a magnetic carrier drug microsphere and bioactive artificial bone under magnetic fields in vitro (OMF and/or SMF) for vascularization has not been reported before.

Basing on those above theories, we constructed superparamagnetic chitosan plasmid (pReceiver‐M29‐VEGF165/DH5a) gelatin microspheres (SPCPGMs) and obtained their optimal formula through a cross‐linking curing method. Then, we poured the SPCPGMs into a porous n‐HA/PA66 scaffold. The filled scaffold was planted in a model of a large segmental radius bone defect in a New Zealand rabbit. The in vitro release of the plasmids in the SPCPGMs was observed in the presence of OMF. in vivo vascularization in the artificial bone was also observed. This application is expected to provide a new type of angiogenesis strategy for bone tissue engineering.

## 2 METHODS

### 2.1 Preparation and characterization of SPCN

Superparamagnetic chitosan nanoparticles (SPCNs) were prepared through a chemical coprecipitation method (Guo, Liu, Hong, & Li, 2010). Specifically, a mixture containing ammonium ferrous sulfate and ammonium ferric sulfate was dissolved with 2% chitosan (w/v) in acetic acid solution (pH 5.5). The resulting solution was designated as Solution A. The solution was transferred into a 500 ml three‐necked flask. For Solution B, 6 mol/L NaOH was prepared. Under the protection of a nitrogen atmosphere, Solution A was heated to 55°C with vigorous stirring. Solution B was added dropwise into Solution A for 10 min. The stirring speed was lowered, and the reaction mixture was heated to 85°C for 90 min. An appropriate amount of glutaraldehyde was added to react for 30 min. The pH of the resulting suspension was adjusted to 5.5 with diluted hydrochloric acid. The obtained black colloidal precipitate was washed with distilled water and dried under vacuum to form a dry powder, which was stored at room temperature. The morphologies of the prepared SPCNs were observed by TEM (Hitachi‐600, Japan), and the diameter and distribution were measured by using a laser diffraction particle size analyzer (Rise‐2008, China). The electric potential was measured by a zeta potential analyzer (Malvern, UK). Nitrogen content in chitosan was determined using a Kjeldahl apparatus (QSY‐2, China). The structural characteristics of the samples were evaluated using FT‐IR (Shimadzu, Japan).

### 2.2 In vitro cell transfection of SPCPNC under SMF

VEGF165 plasmids (pReceiver‐M29‐VEGF165/DH5a) were amplified in large quantities, and a competent‐state DH5α *Escherichia coli* strain was used. The plasmids were extracted, purified, and concentrated with a plasmid extraction kit (OMEGA, GA, USA). We used a SmartSpecTM3000 nucleic acid protein determinator (Bio‐Rad, California, USA)) to determine the concentration of nucleic acid. According to the N/P ratios of 1:4, 1:3, 1:2.5, 1:2, 1:1.5, 1:1.25, 1:1, 1.25/1, 1.5/1, 2.0/1, 2.5/1, and 3.0/1, SPCN was dissolved in 1 ml of sodium acetate buffer solution (pH 5.4), then heated to 55°C. Approximately 0.3 mg/ml of plasmids was added into the solution. The plasmids were then placed under vortex stirring for 20 s and compounded for 1 hr at 55°C. λDNA/HindIII digest was used as marker. The binding status of the plasmids with SPCN was observed by using agarose gel electrophoresis (AGE). The internal structure of the SPCNPC was observed by AFM (AFM‐IPC‐I08B). Under an SMF intensity of 200 mT, 1 × 10^5^ HUVEC‐1, human osteogenic sarcoma (MG‐63) and 293 cells were inoculated into a 12‐pore cell culture plate. The genetic in vitro transfection experiment was conducted after constant‐temperature incubation for 24 hr. Negative control pores were set for both kinds of cells. Three groups of parallel experiments were set for each experimental group. Flow cytometry (FCM; FACScan, Franklin Lakes, USA) was performed to observe cell magnetofection rate. The phagocytosis of SPCNPC in HEK‐293 was observed by TEM (Hitachi‐600, Japan).

### 2.3 Optimized preparation and characterization of SPCPGM under orthogonal design

An orthogonal design was used for the optimization of the parameters for SPCPGM preparation. The cross‐linking curing method was then used for the preparation of SPCPGM with different components. Fe_3_O_4_ proportion (a), plasmid dosage (b), glutaraldehyde dosage (c), and emulsification rotation speed (d) were used as influencing factors. Days of microsphere plasmid release and saturation magnetization were considered comprehensive evaluation indexes. An orthogonal test was conducted according to L9 (34) (Table 1). CPGM was used as control. The procedures of this method were as follows: magnetic chitosan nanoparticles containing different proportions of Fe_3_O_4_ were added to 5 ml of acetic acid buffer (pH 5.5). The resulting mixtures were heated to 55°C. A certain dose of plasmid (pReceiver‐VEGF165/DH5a) was heated to 55°C and then mixed by vortexing for 20 s and subjected to a binding reaction for 1 hr. Approximately 20% of the gelatin solution (w/v) was preheated at 50°C, then added and mixed to form a composite emulsion, which was subsequently added to paraffin oil (50°C) and preheated with a homogenizer (IKA‐T25, Germany) at a proper rotation speed. After confirming the presence of microspheres, we lowered the temperature to 4°C with an ice bath. An appropriate amount of 37% formaldehyde and isopropanol was added, and the resulting mixture was continuously stirred for 1 hr. The samples were washed from five to eight times with ether and deionized water (the washing solution was saved to determine its plasmid content). The microspheres were collected by centrifugation, lyophilized, and stored at −20°C. Superparamagnetic chitosan was replaced with chitosan for the preparation of CPGM.

**Table 1:** Factors and levels of the orthogonal design

Level	Factor			
	A/(%,w/v)	B/(mg)	C/(mg)	D/(rpm)
1	40	8	7.5	300
2	20	4	5.3	400
3	10	2	2.5	500

The magnetic properties of SPCPGM were measured with a BHV‐55 vibrating sample magnetometer (VSM). The morphologies were then investigated by light microscopy (LM; Olympus‐CK‐2, Japan) and scanning electron microscopy (SEM; Hitachi‐S‐3000N, Japan). The diameter and distribution were measured by using a laser diffraction particle size analyzer (Rise‐2008, China). Three batches of microspheres were prepared according to the optimized formula. Scrubbing solutions from all groups were collected and centrifuged (8,000 rpm, 5 min). We collected the supernatants and used the UV method to determine pReceiver‐VEGF165/DH5a plasmid concentration at a 260 nm wavelength (we used a microsphere scrubbing solution without plasmids to set the equipment at zero value and to eliminate interference from auxiliary materials). The weights of these microspheres were measured. The drug‐loading capacity and encapsulation efficiency of microcapsules were calculated according to the following formula: encapsulation efficiency = [(input drug dosage − drug dosage in supernatant)/input drug dosage] × 100%; drug‐loading capacity = [(input drug dosage − drug dosage in supernatant)/microsphere weight] × 100%.

### 2.4 In vitro plasmid release experiment of SPCPGM under OMF

The n‐HA/PA66 composite scaffold provided by the Research Center of Nanobiomaterials in Sichuan University was used for the preparation of porous bone implants (Figure 4a). n‐HA/PA66 scaffolds is a hollow cylinder with a side hole, each having a length of 1.2 cm, inner diameter of 0.30 cm, and outer diameter of 0.40 cm. SPCPGM or CPGM were filled into the porous bone implants (Figure 4b) and plasmid release status was observed under a thermostatic water bath and OMF.

Plasmid release status was observed in three ways as follows: Group A, SPCPGM + OMF, Group B, SPCPGM without application of OMF, and Group C, CPGM + OMF. Two samples in each group were observed. Each microsphere sample weighed 2.00 mg (SPCPGM contained ~1.06 mg of plasmids, and CPGM contained ~1.30 mg of plasmids). The sample was placed into a 25 ml conical flask added with 10 ml of 0.01 M phosphate buffered saline (PBS; pH = 7.4) and then into a thermostatic water bath oscillator (37°C) at an oscillation frequency of 100 times/min.

### 2.5 In vivo experiment of artificial bone vascularization

All animal experiments were approved by the Association for Assessment and Accreditation of Laboratory Animal Care International, China, and the Experimental Animal Committee of the Chongqing Medical University (Permit numbers: SCXK [Yu] 2012‐0001 and SYXK [Yu] 2012‐0001). The methods were performed in accordance with the approved guidelines. We selected 32 New Zealand white rabbits to establish a bone defect model at two sides of a rabbit radius. These rabbits were randomly divided into four groups as follows: Group A: SPCPGM + SMF + OMF; Group B: SPCPGM + SMF; Group C: single application of SPCPGM; and group D: CPGM (blank control). At the 2nd, 4th, 6th, and 8th week after the operation, Su Mian Xin (0.2–0.3 ml/kg) was used as intramuscular anesthetic for the animals. The total radioactive count and average count value were first determined in the bone scaffold region through 99MTc‐MDP radionuclide tomography and then used for analyzing vascularization at the bone scaffold. The blood vessels were perfused and dyed with ink. Finally, the animals were killed by injecting air into their ear veins. The samples were collected, and the status of angiogenesis in the scaffold was observed. Paraformaldehyde (4%) was used to fix the samples, which were then prepared and dyed with HE. Qualitative and preliminary observation of the vascularization was performed under an optical microscope. Newly generated blood vessels were determined qualitatively and quantitatively by using the stereological image generated by the Tiger 920G image analysis software system. The conditions of the newly generated vessels in the four groups, each of which were subjected to different treatments, were evaluated through statistical analysis.

### 2.6 Statistical analysis

SPSS 10.0 software package was used for statistical analysis and data processing. All data were expressed as mean ± standard deviation. Variance analysis (one‐way ANOVA) was used for multigroup comparisons. Statistical analysis of the obtained data was implemented. *p* < .05 indicated that the difference is statistically significant while *p* < .01 indicated that the difference is highly significant.

## 3 RESULTS

### 3.1 Preparing and characterizing SPCNs

**Figure 1:**
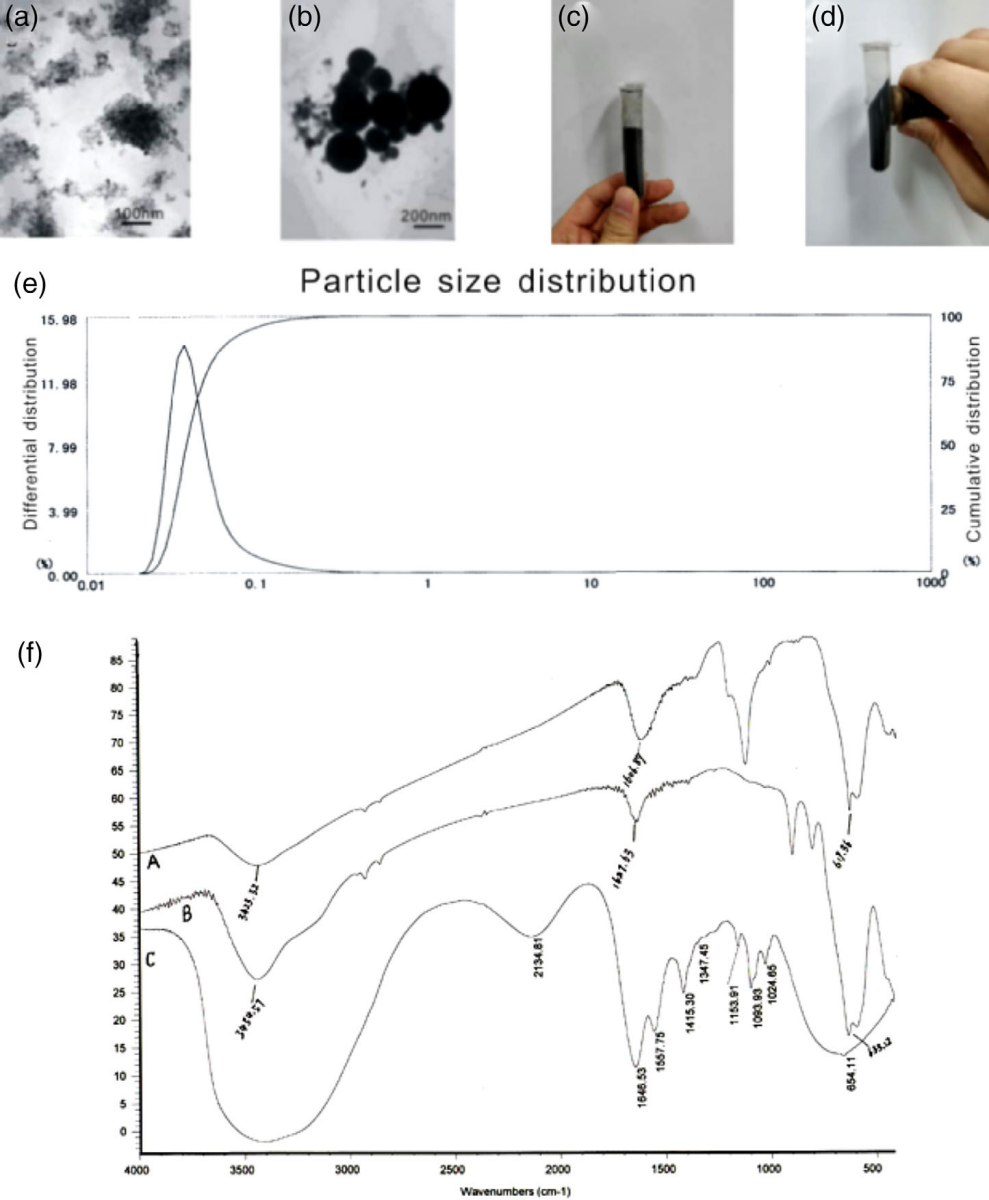
Characterization of superparamagnetic chitosan nanoparticle (SPCN). A, transmission electron microscopy (TEM) photograph of SPCN (×100,000). B, TEM photograph of SPCN (×80,000). C, Fe_3_O_4_ magnetic fluid without a magnetic field (face tilt). D, Fe_3_O_4_ magnetic fluid with a magnetic field (face tilt). E, Granulometer report. The average particle size: 0.046 ± 0.024 μm; cumulative percentage: 10% = 0.029 μm; 50% = 0.039 μm; 90% = 0.069 μm; 97% = 0.116 μm; 99.385% ≤ 0.200 μm; 99.898% ≤ 0.300 μm. Curve fitting coefficient: 0.848. F, FT‐IR spectra: (a) Chitosan, (b) nano‐Fe_3_O_4_, and (c) SPCN

SPCNs were prepared by coprecipitation (Guo et al., 2010). The SPCNs were circular or oval and had favorable evenness, as observed through transmission electron microscopy (TEM; Figure 1A,B). Figure 1C shows the Fe_3_O_4_ magnetic fluid, and Figure 1D shows the directional migration of magnetic fluids under a magnetic field. The average particle size of SPCN was 0.046 ± 0.024 μm (Figure 1E), the electric potential was positive, and the zeta potential on the SPCN surface was 70.5 ± 11 mV. The nitrogen content of SPCN was 0.007 mg/L. Figure 1F shows the Fourier transform infrared spectroscopy (FT‐IR) spectra of chitosan (Figure 1F‐a), Fe_3_O_4_ nanoparticles (Figure 1F‐b), and SPCN (Figure 1F‐c). The absorption peaks of the stretching vibration for the N─H, O─H, incomplete deacetylated carbonyl, pyran ring C─O─C, and pyran ring C─O─C groups were observed at 3,400–3,500, 1646.53, 1557.75, 1024.65, and 1093.93 cm^−1^, respectively (Figure 1F‐a). The Fe_3_O_4_ nanoparticles exhibited a specific absorption peak at 633.12 cm^−1^ (Figure 1F‐b). In the SPCN (Figure 1F‐c), the Schiff base absorption peak was formed by the cross‐linking between glutaraldehyde and chitosan at 1606.89 cm^−1^. The absorption peak from the C─O─C stretching vibration of the chitosan pyran ring at 1,100 cm^−1^ was retained, whereas the specific absorption peak of Fe_3_O_4_ was red‐shifted to 619.36 cm^−1^. The FT‐IR results showed that the specific vibrational absorption peaks of Fe_3_O_4_ crystal and chitosan were present in the SPCN. Chitosan and Fe_3_O_4_ were successfully coated and formed SPCNs with nonvirogene carrier properties suitable for further experiment.

### 3.2 Preparation and in vitro magnetofection of the SPCNPCs

**Figure 2:**
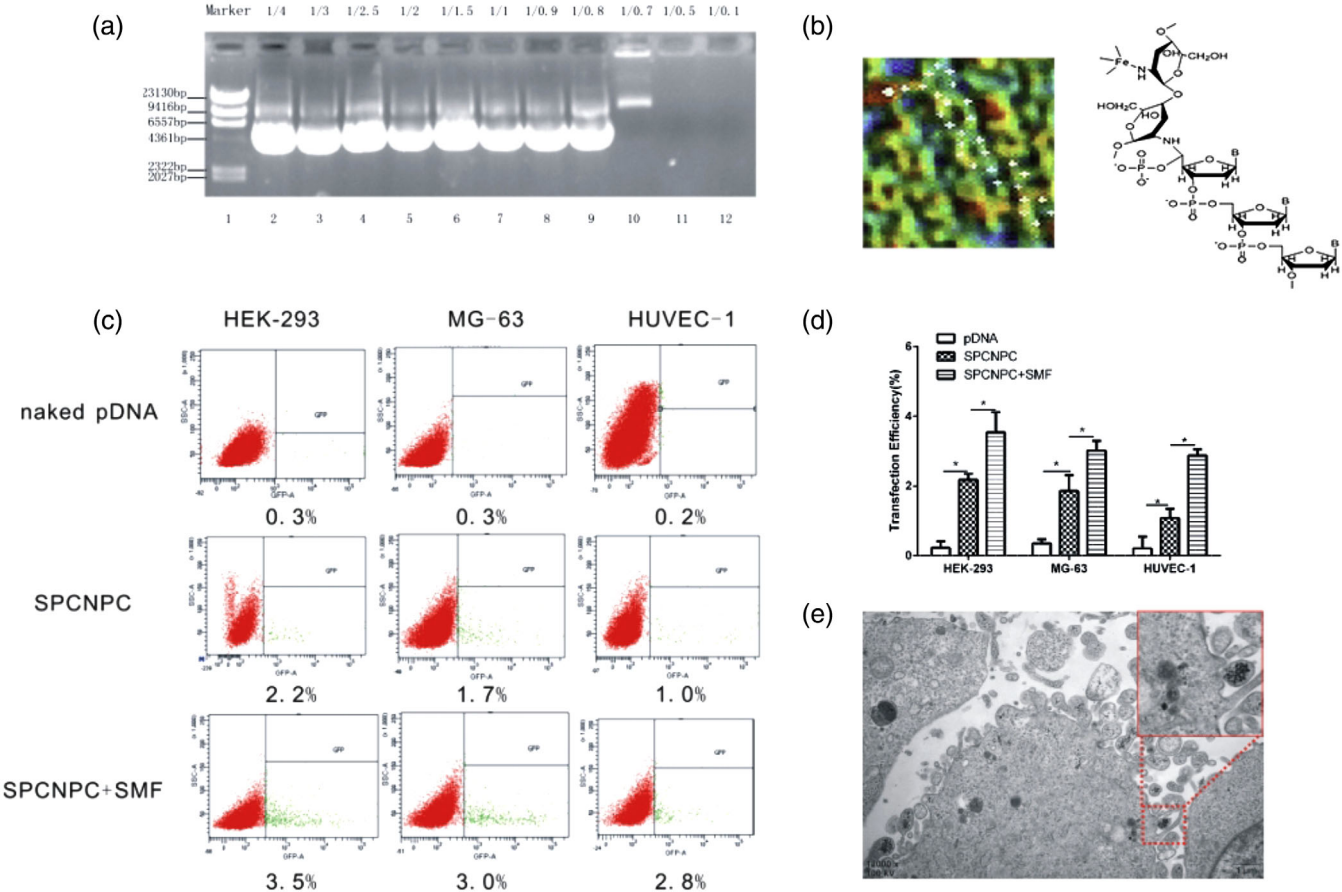
Preparation and in vitro magnetofection of SPCNPC. a, Plasmid binding experiment, marker is λDNA/HindIII digest. b, Atomic power microstructural morphology of SPCNPC. Red dots represent iron atoms. A six‐membered structure of white dots represents chitosan. Five‐membered ring structure of white dots represents plasmids. The black arrow represents the chemical structure diagram of SPCNPC. c,d, Detection of cell transfection efficiency through flow cytometry (FCM). Error bars represent the standard deviation of the measurements performed on the three samples. *p < .05. e, Phagocytosis of SPCNPC in HEK‐293 under transmission electron microscopy (TEM; ×12,000)

The results of AGE analysis and identification showed that the increase in nitrogen/phosphorus ratio (N/P) gradually weakened the brightness of the specific bright strips of the plasmids (Figure 2a). Thus, plasmids escaping from the spotting holes gradually decreased. At the N/P ratio of 1/0.5 (electrophoresis channel 11), no specific bright strip occurred, indicating that the SPCN had completely bound with the plasmids and were thus unable to escape from the spotting holes. The zeta potential of SPCNPC was 27.8 ± 4 mV. These results laid a foundation for the subsequent in vitro transfection experiment. Figure 2b shows the atomic power microstructural morphology of SPCNPC. The morphology was in line with the expected chemical structure diagram (denoted by the black arrow). This result indicates that chitosan had been successfully modified to the Fe_3_O_4_ nanoparticles and can successfully bind the DNA.

The transfection efficiency of SPCNPC in the epithelial cells of a human kidney (HEK‐293), bone formation sarcoma cells (MG‐63), and umbilical vein endothelial cells (HUVEC‐1) under SMF was determined by flow cytometry (FCM; Figure 2c,d). The SPCNPC + SMF had the highest green fluorescent protein expression level in HEK‐293 cells, followed by SPCNPC and naked pDNA. This result was consistent with those obtained from the HUVEC1 and MG‐63 cells. The transfection rates of the naked plasmids in the HEK‐293, HUVEC‐1, and MG‐63 cells were 0.23% ± 0.18%, 0.35% ± 0.12%, and 0.21% ± 0.34%, respectively. After the plasmids were coated into the SPCNs, the transfection rates of the plasmids increased to 2.18% ± 0.17% (*p* < .05), 1.86% ± 0.45% (*p* < .05), and 1.08% ± 0.27% (*p* < .05). Under SMF, the transfection rates further increased to 3.54% ± 0.58% (*p* < .05), 3.02% ± 0.2% (*p* < .05), and 2.87% ± 0.19%. The results suggest that SMF promotes the transfection of SPCNPCs and its transfection efficiency can be increased by 2–3 times. Figure 2e shows that SPCNPC was devoured by HEK‐293, and its ultrastructure did not show obvious changes. An instance of phagocytosis can be observed inside the red dotted line. Owing to the pull of the SMF, SPCNPC can closely come into contact with the cells in a short period of time. Thus, the local concentration of DNA increased, and DNA transfection was accelerated.

## 4 OPTIMIZED PREPARATION OF SPCPGMS

Table 2 lists the results of variance analysis performed during the orthogonal experiment. According to the pre‐experiment and experimental objective in the early stage, the comprehensive score was obtained by adding the number of plasmid released daily to the value of saturation magnetization (total scores = number of plasmid release days + saturation magnetization). The range values reflected the degrees of influence of various factors on different indicators. The greater the value is, the greater the degree of influence is. The degrees of influence of the four factors on aggregate indicators were in the following order: A > B > C > D. The best level was selected from these factors and the optimal combination was A2B2C2D3. Specifically, the amount of Fe_3_O_4_, plasmid, and glutaraldehyde were 20% (w/v), 4 mg (w/v), and 5.3 mg (w/v), respectively. The emulsification rotation speed was 500 rpm.

**Table 2:** Results of the orthogonal design

Number of experiments	A/(%,w/v)	B/(mg)	C/(mg)	D/(rpm)	Time(day)	MS(emu/g)
Experiment 1	40	8	7.5	300	17	11.223
Experiment 2	40	4	5.3	400	18	11.965
Experiment 3	40	2	2.5	500	15	10.741
Experiment 4	20	8	5.3	500	22	8.892
Experiment 5	20	4	2.5	300	21	8.013
Experiment 6	20	2	7.5	400	17	7.658
Experiment 7	10	8	2.5	400	10	4.061
Experiment 8	10	4	7.5	500	11	3.922
Experiment 9	10	2	5.3	300	8	4.024
K1	83.929	73.176	67.803	69.26	/	/
K2	84.563	73.9	72.881	68.684	/	/
K3	41.007	62.423	68.815	71.555	/	/
R	14.5187	3.8257	1.6927	0.957	/	/

## 5 VERIFICATION OF OPTIMIZED PRESCRIPTION

**Figure 3:**
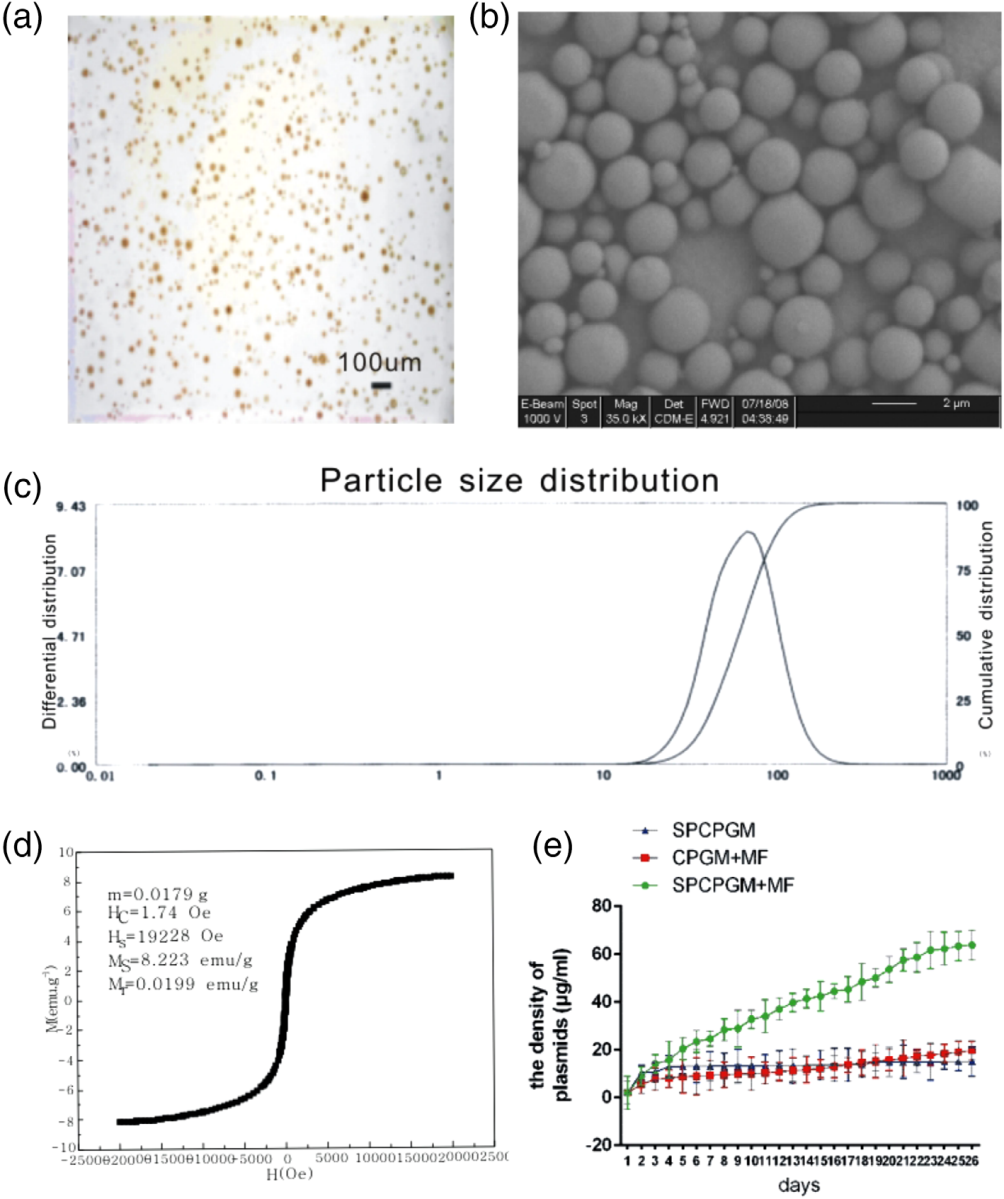
Verification of optimized prescription. a, light microscopy (LM) photograph of SPCPGM. (×400). b, scanning electron microscopy (SEM) photograph of SPCPGM. (×35,000). c, Granulometer report. The average particle size: 65.358 ± 20.931 μm; cumulative percentage: 10% = 33.521 μm; l50% = 60.198 μm; 90% = 103.656 μm; 97% = 132.523 μm; 0.000% ≤ 0.200 μm; 0.000% ≤ 0.300 μm Curve fitting coefficient: 0.921. d, Hysteresis loop. e, in vitro plasmid release experiment for SPCPGM

Three batches of SPCPGM samples were prepared by using the optimal results shown in Table 1 and observed through LM and SEM. Most of the microspheres were round and spherical and had concave–convex surfaces, uniform size, and favorable dispersity (Figure 3a,b). The average particle size was 65.358 ± 20.931 μm (Figure 3c). Figure 3d shows the hysteresis loop of SPCPGM at room temperature. The magnetization of the samples approached saturation values when the applied magnetic field was increased to 25,000 Oe. The saturation magnetization of SPCPGM was 8.223 emu/g. A small remnant magnetization of 0.0199 emu/g was obtained at the external magnetic field of 0 Oe, indicating that the magnetic particles produced were superparamagnetic. The results show that the encapsulation efficiency and drug‐loading capacity of SPCPGM were 98.51% ± 1.34% (w/w) and 53.42% ± 1.54% (w/w), respectively.

OMF was used in the in vitro plasmid release experiment for the SPCPGMs. OMF was applied to group A because the daily drug release was ~4–6 μg at the 1st day of the experiment. The sustainable release quantity of Group A was apparently higher than those in the other two groups (~four times). Results of the statistical variance analysis showed that Group A was significantly different from Groups B and C in terms of drug dissolving‐out quantity (Q test, *p* < .001). The difference between Groups B and C in drug‐release quantity was nonsignificant (Q test, *p* > .05).

The dissolution rate of Group A from the 22nd to the 25th days of the experiment slowed down and drug‐release percentage of the plasmids was ~70%. The approximate values of the drug‐release percentage of the plasmids in the groups not subjected to OMF were 11% in SPCPGM and 15% in chitosan plasmid (pReceiver‐M29‐VEGF165/DH5a) gelatin microspheres (CPGMs; Figure 3e).

## 6 IN VITRO EXPERIMENT OF VASCULARIZATION OF ARTIFICIAL BONE

**Figure 4:**
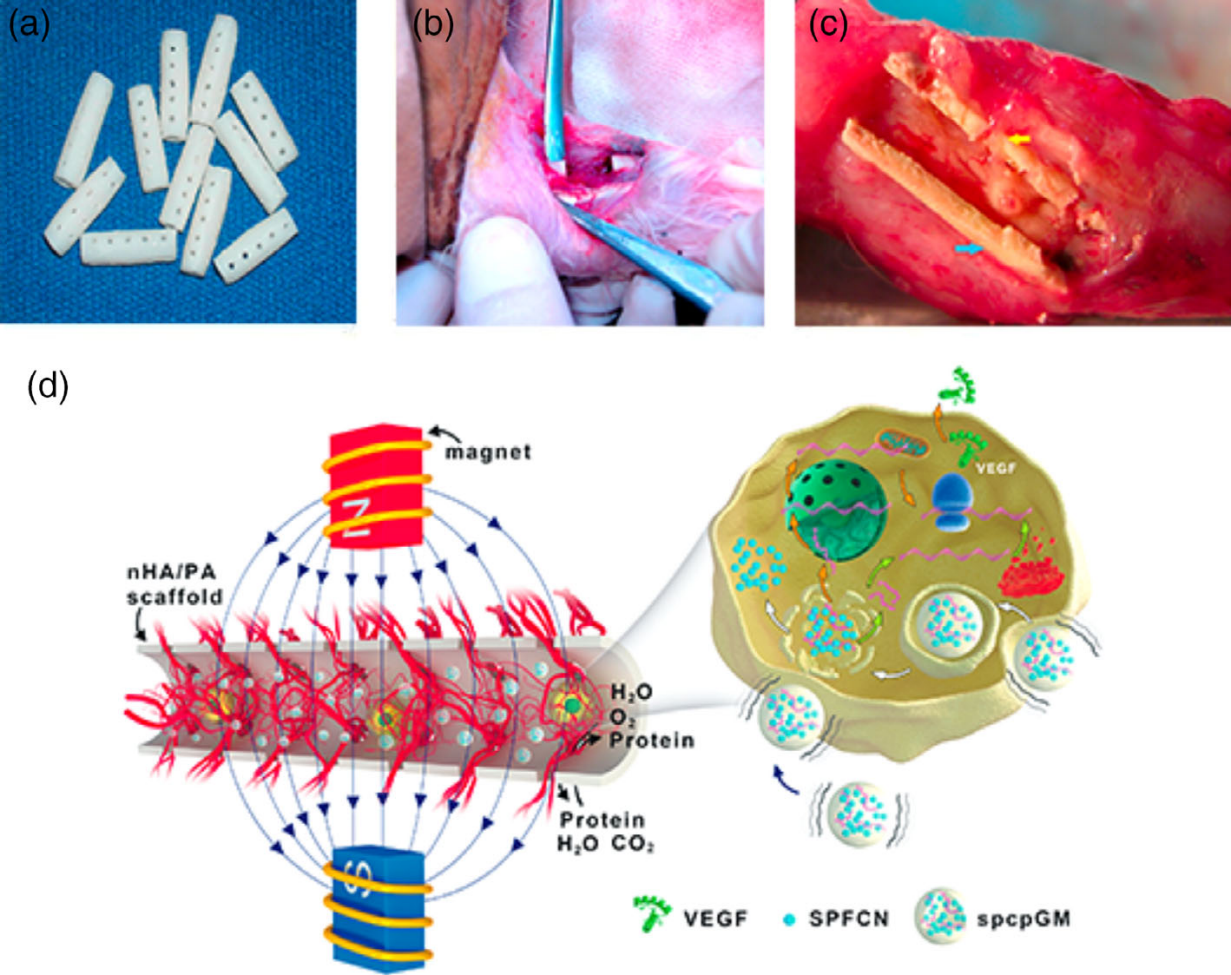
Repair of a radius defect in a New Zealand white rabbit with an artificial bone scaffold loaded with SPCPGM. a, n‐HA/PA66 artificial bone scaffold. b, A model of a large segmental radius bone defect was established in New Zealand rabbits. c, General observation at 6 weeks postoperation. The blue arrow represents the artificial bone scaffold. The yellow arrow shows that granulation tissues grow into the side holes of the artificial bone implants. d, Schematic diagram of SPCPGM facilitating vascularization inside the artificial bone scaffold

An artificial bone scaffold in a hollow structure with lateral holes was prepared by using n‐HA/PA66 bone cement with good histocompatibility (Figure 4a). Then, the SPCPGMs was loaded into the hollow portion of the scaffold. A model of a large segmental radius bone defect was established in New Zealand rabbits (Hou et al., 2015; Figure 4b), and an artificial bone scaffold loaded with SPCPGM was implanted. Lastly, the angiogenesis results of the artificial bone scaffold were observed under different magnetic fields.

## 7 OBSERVATION OF GROSS MORPHOLOGY

On the 1st day after the operation, experimental animals started eating and performing their activities. A week later, all the animals completely restored their normal eating activities and performed other activities. Their incisions gradually healed without infection 2 weeks later. When the animals bit the fixed magnets at their upper limbs, the magnets were fixed immediately. After one animal accidentally died, immediate supplementation was made. The other animals survived until the preset sampling date.

On the 2nd week, the implants in Group A were wrapped by loose fiber tissues. A small quantity of vascularized soft tissues grew into the side holes of the implants. One‐third of the microspheres formed residues in the hollow parts of the implants. In Group B, the implants were wrapped by loose fiber tissues without obvious neovascularization. In Groups C and D, half of the microspheres formed residues in the hollow parts of the implants wrapped by a small amount of fiber tissues. The residues of the microspheres in the hollow parts of the implants was ~1/2.

On the 4th week, the implants in Group A were wrapped by fiber tissues. A large amount of vascularized soft tissues grew into the side holes of the implants, and a small amount of microsphere residue was observed in the hollow part. In Group B, a small amount of vascularized soft tissues grew into the side holes of the implants, and the microsphere residue in the hollow part was ~1/3. In Groups C and D, a small amount of vascularized soft tissues grew into the side holes of the implants and microsphere residue in the hollow part of the implants was ~1/3.

On the 6th week, the implants in Group A were completely wrapped by compact fiber membranous tissues and a large quantity of vascularized soft tissues grew into the side holes of the implants(Figure 4c). In Group B, vascularized soft tissues grew into the side holes of the implants. Meanwhile, a large amount of vascularized soft tissues grew in the side holes of the implants in Groups C and D.

On the 8th week, the implants in Group A were completely wrapped by compact fiber membranous tissues, and a large amount of vascularized soft tissues grew into the side holes of the implants. Regenerated tissues were also dyed into black after ink perfusion. In Group B, vascularized soft tissues grew into the implant side holes. Similarly, vascularized soft tissues grew into the side holes of the implants in Groups C and D but in large amounts.

Figure 4d shows the schematic diagram of the SPCPGM mechanism involved in artificial bone scaffold vascularization. Superparamagnetic nanoparticles subjected to magnetic fields generated magnetic micromotion, which facilitated the local interchange of nutrients and entry of nutrients, such as protein, and oxygen into the scaffold while discharging metabolite. SPCPGM subjected to OMF released positively charged chitosan plasmid compounds, which adhered to the negatively charged cytomembrane enter before entering the cells. Angiogenesis was facilitated through the transcription, translation, and expression of the VEGF protein.

## 8 HISTOLOGICAL OBSERVATION

**Figure 5:**
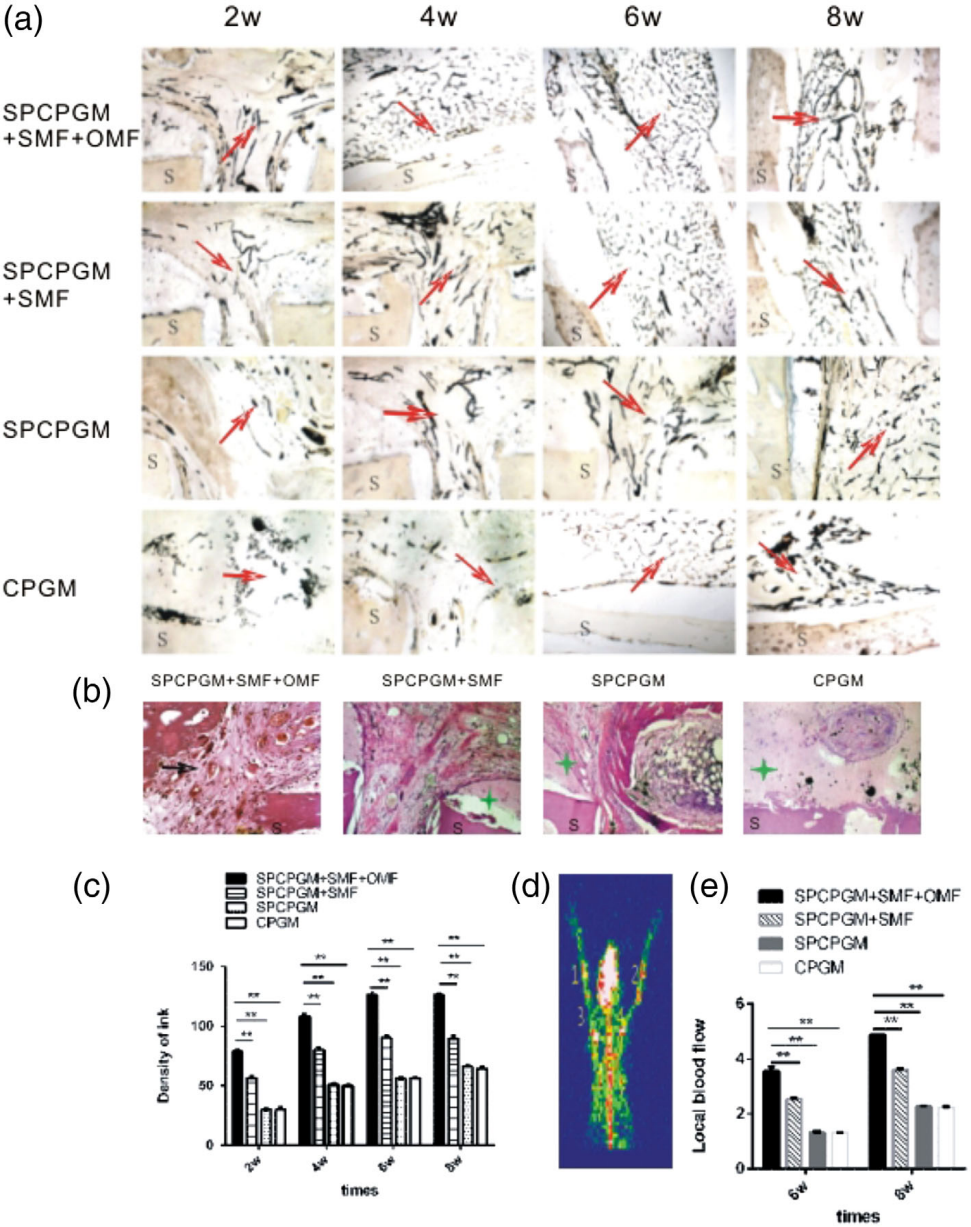
In vivo experiment of SPCPGM facilitating artificial bone vascularization under magnetic fields (OMF and/or SMF). a, Ink dyeing for rabbit radius implant (×40). S is the n‐HA/PA66 artificial bone scaffold. The red arrow shows the blood vessel dyed after ink perfusion. b, Hematoxylin and eosin staining (×40). S is the n‐HA/PA66 artificial bone scaffold. The green asterisk represents residual microspheres. The black arrow points at new vessels. c, Variance analysis of ink dyeing after operation. The data are expressed as the means ± standard deviation (*SD*) of 64. ^**^ *p* < .001. d, Radionuclide tomography. The pictures show a collection of radionuclides of Group A in the sixth week. e, Average radioactive count of radionuclide blood flow phase. The data are expressed as the means ± *SD* of 64. ^**^ *p* < .001

The samples were dyed with ink (Figure 5a) and hematoxylin and eosin (HE; Figure 5b). In Group A, the connective tissues grew around the implants and into the lumens and were infiltrated by a number of inflammatory cells 2 weeks after the operation. Immature blood capillaries were also observed. The ink‐perfused blood vessels formed net‐like structures. Few soft cartilages grew inward, and microsphere residues were occasionally observed. In the 6th week, mature blood vessels formed and fibrous porosis occurred inside the scaffold. Nearly no microsphere residue was observed. In the 8th week, mature vascular nets and soft bones filled the lumen. Vascularization in Group B was similar to that in Group A but occurred 2 weeks later than that in Group A. A large amount of microsphere residues was observed in the 8th week. Vascularization times in Groups D and C were longer than that of Group B, and large nondegraded microsphere residues were observed. Figure 5c shows a stereological image analysis of the ink‐dyed picture obtained through Tiger 920G image analysis software. From the 2nd to the 8th week, Group A had the highest blood vessel density among the groups. Variance analysis (*Q* test) showed significant differences (*p* < .01). Blood vessel density in Group B was significantly higher than those in Groups C and D (*p* < .01). Multiple comparison test results (post hoc) indicated that the difference between Groups C and D was not statistically significant (*p* > .05). As shown in Figure 5d, blood flow signals were present in the bone defects (1, 2, 3, and 4). The average radiation counts (counts/pixel) of radionuclide blood flow were observed. From the 6th to the 8th week, Group A had the highest local blood flow volumes (count values) in the bone defects. Meanwhile, the result in Group C was approximate to that in Group D, and their local blood flow volumes (count values) were low (Figure 5e).

## 9 DISCUSSION

The three basic processes after bone transplantation are implant vascularization, osteogenesis, and epiphysis fusion (Gross, Cox, & Jinnah, 1993). The key link is vascularization, which occurs during transplantation and restoration. Notably, vascularization remains to be the foundation of bone defect restoration after the transplantation of bioactive artificial bone (Almubarak et al., 2016). Sufficient blood supply is a crucial factor for the successful in vivo transplantation of bioactive artificial bone tissues. The dispersion and permeation of nutritive substances can only reach 150–200 μm around blood vessels (Lovett et al., 2009). Therefore, determining the possibility of rapid blood supply reconstruction after artificial bone transplantation remains a challenge when bioactive artificial bone research is applied to clinical settings.

Using all kinds of cell growth factors and combining them with scaffold materials for angiogenesis are among the main reconstruction strategies for supplying blood in artificial bones (Lindhorst, Tavassol, von, et al., 2010; Lovett et al., 2009; Sun et al., 2013). The other main strategies include using transgenic cells to construct bone tissues (Kawai et al., 2017; Kawai, Bessho, Maruyama, Miyazaki, & Yamamoto, 2006), using tissues that contain abundant vascular nets and wrapping or implanting them into a bone scaffold material (Li & Kawashita, 2011; Türer & Önger, 2017; Wu et al., 2018), using a drug (gene) release system for the vascularization of artificial bones or bone tissues, and performing vascularized bone tissue preconstruction (Hall, 2007; Lan, Tian, ZhuGe, et al., 2017; Moncion, Lin, O'Neill, et al., 2017). Bioactive artificial bones containing magnetic drug‐carrying microspheres that facilitate vascularization under in vitro magnetic field (SMF or OMF) is currently unreported.

Therefore, we propose the application of an in vitro noninvasive OMF and SMF for vascularization. This approach promotes the micromovement of magnetic gene‐loaded microspheres inside an artificial bone scaffold. SPCPGMs were constructed, and the optimal formula was obtained through a cross‐linking curing method. The porous n‐HA/PA66 scaffold was then filled with SPCPGM, and the resulting complex was planted in large segmental radius bone defects in New Zealand rabbits. The in vitro release of the plasmid in SPCPGM was observed under OMF.

According to the retrieved documents (Denkbas, Kilicay, Birlikseven, et al., 2002; Guo et al., 2010), the following data were obtained from magnetic microsphere preparation: gelatin proportion of ~25%, temperature of 55°C, which decreased to 4°C after an ice bath, stirring rate of 300–500 r/hr, and cross‐linking agent dosage of 200 μl/20 min. Magnetic microspheres with the required sizes were obtained. Meanwhile, as determining the proportions of magnetic nanoferroferric oxide and plasmids in the formula and dosage of a curing agent is necessary, an orthogonal experiment based on single‐factor investigation was designed. The quantity of released microsphere plasmids per day, lasting day, and saturation magnetization were considered as comprehensive evaluation indexes. Meanwhile, the proportion of Fe_3_O_4_ content (a), plasmid dosage (b), glutaraldehyde dosage (c), and emulsification rotation speed (d) were the main factors. The following optimal microsphere preparation formula was obtained by investigating these factors: Fe_3_O_4_ dosage, 20% (w/v); plasmid dosage, 4 mg; glutaraldehyde dosage, 5.3 mg; and emulsification rotation speed, 500 rpm. Under OMF, the daily quantity of plasmid released reached 4–6 μg for 21 days. These plasmids were placed into the hollow scaffold of the artificial bone implant for the in vitro drug release experiment. The effect of OMF on plasmid release in SPCPGM was then observed. In addition, SPCPGM and CPGM without OMF were set as controls. The dissolution rate of the release system in SPCPGM + OMF group decreased from the 22nd to the 25th day of the experiment. The drug release rate of plasmids was 70%. The approximate values of drug release rates of plasmids in groups without OMF were all 11% SPCPGM and CPGM. Therefore, plasmid release in SPCPGM and CPGM is a long‐term process, and OMF can facilitate plasmid dissolution from porous bone implant containing SPCPGM. This mechanism increases the original plasmid dissolving‐out quantity four‐ or fivefold in the 3rd week.

The results of the in vivo experiment indicated that the effect of SPCPGM on vascularization inside the scaffold can be improved under oscillating and static magnetic fields. Moreover, the extent of vascularization reached its peak in the 6th week. The SPCPGM released positively charged chitosan plasmid compounds that bind to negatively charged cytomembranes. The release facilitated the entry of plasmids into the cells. Meanwhile, angiogenesis was promoted through the transcription, translation, and expression of the VEGF protein. However, the dispersion and permeation of nutritive substances inside the scaffold only reached 150–200 μm from the blood vessel. In this experiment, the blood vessel was allowed to grow into the scaffold with insufficient supply of nutrient substances. The specific mechanism remains to be clarified, and whether the magnetic transfection of cells was completed because OMF facilitated the interchange of oxygen, protein, and other nutritive substances inside and outside the scaffold and surrounding cells still needs to be verified through a follow‐up experiment.

Under external magnetic fields, this system has realized three major functions, namely, sustained plasmid gene release inside the artificial bone scaffold, magnetic plasmid gene transfection of local cells, and interchange of nutritive substances through magnetic micromotion inside the scaffold. The highly efficient principles involved in the interchange of nutritive substances through magnetic micromotion, gene release, and genetic transfer under external magnetic fields were proposed and are expected to be useful in discovering novel strategies for angiogenesis and in bone tissue engineering research.

## 10 CONCLUSIONS

The optimal preparation formula and technology of SPCPGM were as follows: Fe_3_O_4_ dosage, 20% (w/v); plasmid dosage, 4 mg (w/v); glutaraldehyde dosage, 5.3 mg; and emulsification rotation speed, 500 rpm. Daily release of plasmids by optimized SPCPGM under OMF was 4–6 μg for 3 weeks. These conditions are conducive to vascularization inside artificial bone scaffolds. The in vivo animal experiment confirmed that the joint action of SMF and OMF improved the effect of optimized SPCPGM on vascularization inside the scaffold, especially on the 6th week. Therefore, magnetic micromotion and interchange of nutritive substances inside a large segmental artificial bone scaffold can form a highly efficient system that enables the interchange of nutritive substances, magnetic micromotion, genetic release, and genetic transfer under external magnetic fields.

This system is expected to become a new strategy for the vascularization of various in vivo implants. The system enables the volume of artificial tissue engineering implants to reach the standard size range for clinical applications. We surmised that this method can be extensively used in restoring defects and treating large segmental bone tissues.

## DISCLOSURE

No potential conflict of interest was reported by the authors.
